# Three-Dimensional Breast Cancer Model to Investigate CCL5/CCR1 Expression Mediated by Direct Contact between Breast Cancer Cells and Adipose-Derived Stromal Cells or Adipocytes

**DOI:** 10.3390/cancers15133501

**Published:** 2023-07-05

**Authors:** Martin Watzling, Lorenz Klaus, Tamara Weidemeier, Hannes Horder, Regina Ebert, Torsten Blunk, Petra Bauer-Kreisel

**Affiliations:** 1 Department of Trauma, Hand, Plastic and Reconstructive Surgery, University Hospital Würzburg, 97080 Würzburg, Germany; watzling_m@ukw.de (M.W.); lorenz@klaus-bayreuth.de (L.K.); tamara.weidemeier@stud-mail.uni-wuerzburg.de (T.W.); horder_h@ukw.de (H.H.); blunk_t@ukw.de (T.B.); 2 Department of Musculoskeletal Tissue Regeneration, Julius-Maximilians-Universität Würzburg, 97074 Würzburg, Germany; r-ebert.klh@uni-wuerzburg.de

**Keywords:** 3D breast cancer model, adipose-derived stromal cells, adipocytes, adipose tissue, spheroids, co-culture

## Abstract

**Simple Summary:**

In breast cancer, adipose-derived stromal cells (ASCs) and adipocytes, as components of the mammary fat pad, come into close contact with tumor cells. To adequately mimic direct cell–cell interactions between tumor and adjacent stromal cells, a 3D co-spheroid model was developed consisting of ASCs or adipocytes and breast cancer cells (MDA-MB-231, MCF-7). Direct contact between MDA-MB-231 tumor cells and ASCs or adipocytes in this model promoted the expression of C-C motif chemokine ligand 5 (CCL5) and specifically the corresponding receptor C-C chemokine receptor type 1 (CCR1). This, in turn, enhanced the migration of triple-negative MDA-MB-231 breast cancer cells. Such tumor-specific markers up-regulated upon cell–cell contact with adjacent stromal cells may represent promising targets for the detection and treatment of aggressive breast cancer.

**Abstract:**

The tumor microenvironment (TME) in breast cancer is determined by the complex crosstalk of cancer cells with adipose tissue-inherent cells such as adipose-derived stromal cells (ASCs) and adipocytes resulting from the local invasion of tumor cells in the mammary fat pad. This leads to heterotypic cellular contacts between these cell types. To adequately mimic the specific cell-to-cell interaction in an in vivo-like 3D environment, we developed a direct co-culture spheroid model using ASCs or differentiated adipocytes in combination with MDA-MB-231 or MCF-7 breast carcinoma cells. Co-spheroids were generated in a well-defined and reproducible manner in a high-throughput process. We compared the expression of the tumor-promoting chemokine CCL5 and its cognate receptors in these co-spheroids to indirect and direct standard 2D co-cultures. A marked up-regulation of CCL5 and in particular the receptor CCR1 with strict dependence on cell–cell contacts and culture dimensionality was evident. Furthermore, the impact of direct contacts between ASCs and tumor cells and the involvement of CCR1 in promoting tumor cell migration were demonstrated. Overall, these results show the importance of direct 3D co-culture models to better represent the complex tumor–stroma interaction in a tissue-like context. The unveiling of tumor-specific markers that are up-regulated upon direct cell–cell contact with neighboring stromal cells, as demonstrated in the 3D co-culture spheroids, may represent a promising strategy to find new targets for the diagnosis and treatment of invasive breast cancer.

## 1. Introduction

Breast cancer is the most commonly diagnosed cancer in the world, with an estimated 2.3 million new diagnoses in 2020 [1]. Although therapies and treatment approaches have been advanced in recent years, it remains a major health concern. For the development of treatment options, it is pivotal to understand the pathophysiological mechanisms leading to tumor development and metastasis. A key to understanding is not only knowledge about intrinsic anomalies of tumor cells themselves, but also deciphering their complex interaction with cells in the surrounding tissue, the tumor microenvironment (TME) [2]. In the context of breast cancer, the interaction between adipose tissue and breast cancer cells is particularly relevant, as up to 56% of the human non-lactating breast consists of subcutaneous fat compartments. Invasive breast carcinoma readily infiltrates into adjacent adipose tissue and in this way comes into direct contact with tissue-inherent cells such as adipocytes and adipose-derived stromal cells (ASCs) [3,4,5,6]. These interactions have recently been recognized as drivers of cancer malignancy, but may also present promising targets for improved and more personalized diagnosis and therapy options if specific biomarkers involved in these processes can be identified [7,8]. Tumor cell-induced changes and interactions within the TME are multifaceted and driven by indirect paracrine crosstalk between cells, which is mediated by the diffusion of soluble factors. Insights into this were mainly obtained in studies using indirect 2D co-cultures in transwells or conditioned media [9,10,11]. But stromal cells in the TME can also provide locally acting cues triggered by direct heterotypic cell–cell contacts with cancer cells, which are also assumed to play an important role in cancer progression and metastasis formation [12,13]. Unraveling the impact of direct cell–cell interactions in the cancer TME is hampered by the lack of appropriate in vitro models that mimic physical cell–cell contact, ideally in a 3D culture platform. In vivo, cells are closely packed in a 3D environment, in contrast to 2D cell cultures where cell–cell contacts are limited and cellular crosstalk is insufficient. Therefore, in standard 2D cell culture, relevant cell–cell interactions, or the effects thereof, may remain unrecognized [14].

Spheroids represent a valuable tool in this regard as these multicellular aggregates enable close cell contacts in a 3D manner. Multicellular tumor spheroids have been widely employed as an established and well-acknowledged 3D model in preclinical cancer research. To reflect the interaction with cells in the TME, heterotypic spheroid models composed of tumor cells and cells of the TME are increasingly being developed and studied, mostly using fibroblasts as co-cells [15,16]. In order to address the direct interaction between ASCs or adipocytes and breast cancer cells (MDA-MB-231, MCF-7) in a physiologically relevant set-up, an advanced 3D co-culture spheroid model was developed in this study.

C-C motif chemokine ligand 5 (CCL5), also known as RANTES (Regulated on Activation, Normal T cell Expressed and Secreted), has been described as a metastasis-promoting chemokine that is expressed upon the crosstalk between breast cancer and stromal cells [17,18]. Local CCL5 expression was further shown to be elevated in invasive breast carcinoma in peritumoral tissue and was positively correlated with lymph node and distant metastases, underlining the tumor-promoting character of this chemokine [19,20,21]. Findings resulting from in vitro studies, which have so far only been carried out in standard 2D culture, suggest the necessity of close physical cell contacts for CCL5 expression; however, results have been inconsistent [9,17,18,22,23]. The corresponding CCL5 receptors are part of the C-C chemokine receptor (CCR) family and include CCR1, CCR3, and CCR5 [24]. These G protein-coupled receptors have been reported to be involved in CCL5-mediated tumor progression and invasion to varying extents depending on the particular context and cancer type [19,25,26].

We used our newly established 3D co-spheroid model to elucidate the expression of CCL5 and its receptors mediated by ASCs or adipocytes in breast cancer in a more in vivo-like 3D context and compared them to conventional 2D co-culture systems in order to evaluate the impact of the direct 3D co-culture. CCL5 expression was demonstrated to be dependent on direct cellular interaction and culture dimensionality of co-cultures of MDA-MB-231 tumor cells and adipose-tissue derived cells (ASCs or adipocytes), and was furthermore shown to be associated with specific up-regulation of the CCR1 receptor. Migration assays were performed to analyze the functional relevance of the CCL5/CCR1 axis and revealed a migration-promoting role of this specific interaction in triple-negative breast cancer cells.

## 2. Materials and Methods

### 2.1. Cell Culture

Human adipose-derived stromal cells (ASCs) were purchased from Lonza (Basel, Switzerland) and breast cancer cell lines MDA-MB-231 and MCF-7 were obtained from the American Type Culture Collection (ATCC, Manassas, VA, USA). All cells were expanded and cultured in growth medium (Dulbecco’s Modified Eagle’s Medium/Ham’s F-12 (DMEM/F12) *w*/*o* phenol red (Life Technologies, Carlsbad, CA, USA), supplemented with 10% fetal bovine serum (FBS; Life Technologies) and 1% penicillin/streptomycin (100 U/mL penicillin, 0.1 mg/mL streptomycin; Life Technologies) at 37 °C and 5% CO_2_. For the expansion of ASCs, 3 ng/mL basic fibroblast growth factor (bFGF; BioLegend, London, UK) was added to the growth medium. The medium was exchanged every other day. At 80–85% confluence, cells were passaged using 0.25% trypsin-EDTA solution (Life Technologies). ASCs at passage 6 were used for subsequent experiments.

### 2.2. Spheroid Formation and Co-Culture with ASCs

**Figure 1:**
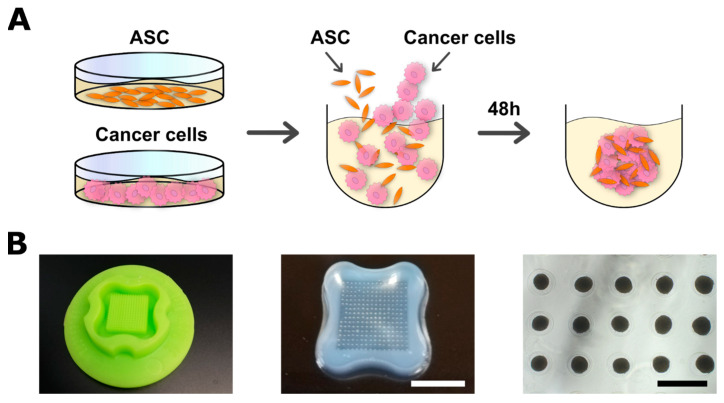
Spheroid formation for co-cultures of ASCs and cancer cells. (**A**) Schematic illustration of spheroid formation. Expansion of cells was performed in 2D before detachment and seeding in agarose micromolds. Cells were permitted to form spheroids over 48 h before harvest. (**B**, left) Silicone profile to cast micromolds. (**B**, middle) Agarose micromolds with spheroids after 48 h (256 spheroids/mold). Bar equals 1 cm. (**B**, right) Microscopic image of spheroids in micromolds after 48 h (1000 cells/spheroid; stromal cells and tumor cells 1:1). Bar equals 500 µm.

Spheroid formation of mono- and co-spheroids was performed using agarose molds, which were cast in MicroTissues^®^3D Petri Dishes^®^ (16 × 16 arrays, Sigma-Aldrich, St. Louis, MO, USA) according to the manufacturer’s instructions (Figure 1). Here, 2.56 × 10^5^ cells were seeded per agarose mold (one agarose mold per well in a 12-well plate) and cultured in growth medium containing 10% FBS and 1% penicillin/streptomycin for two days, resulting in 256 multicellular spheroids per agarose mold (1000 cells/spheroid). Monocultures consisted of 1000 cells of the respective cell types, while co-cultures contained 500 ASCs and 500 cells of either MDA-MB-231 or MCF-7. For direct 2D co-cultures, 1 × 10^4^ cells/cm^2^ of ASCs and breast cancer cells were seeded in a 6-well plate. For indirect 2D co-culture, a transwell system (Greiner Bio-One, Frickenhausen, Germany) was used, in which 1 × 10^4^ ASCs/cm^2^ were seeded in the lower chamber and the same amount of breast cancer cells in the upper chamber. Accompanying all co-cultures, respective monoculture controls were conducted. Cells were cultured for 48 h before harvest. For imaging, ASCs and tumor cells were pre-stained with PKH26 (for tumor cells) and PKH67 (for ASCs, both Sigma-Aldrich) prior to spheroid culture according to the manufacturer’s instructions. Whole mount samples were imaged using an LSM780 confocal microscope (Zeiss, Jena, Germany).

### 2.3. Adipogenic Differentiation of ASCs and Spheroid Formation with Adipocytes

For the generation of multicellular spheroids with adipocytes, ASCs were cultured in adipogenic differentiation medium consisting of growth medium with insulin (final concentration 1.7 µM; PromoCell, Heidelberg, Germany), dexamethasone (1 µM; Sigma-Aldrich), 3-isobutyl-1-methylxanthine (IBMX, 500 µM; Serva-Electrophoresis, Heidelberg, Germany), and indomethacin (200 µM; Sigma-Aldrich). Cells were differentiated in conventional 2D culture for 14 days prior to detachment and subsequent spheroid formation. Successful differentiation was verified by qRT-PCR of adipogenic marker genes and determination of intracellular triglyceride content. Adipocyte-containing mono- and co-spheroids were generated as described above for ASCs.

### 2.4. Characterization of Spheroids

After seeding cells in agarose molds, images of the cell aggregates were acquired every 12 h over a total of 48 h. Diameter was determined using CellSens™ 4.1 software (Olympus, Hamburg, Germany). Roundness was assessed by applying the IsoData threshold and converting photos to binary images, followed by the calculation of values [27], all implemented in a custom macro for ImageJ.

### 2.5. Magnetic-Activated Cell Sorting (MACS) of Co-Spheroids

To be able to differentially assess gene expression of both cell types after co-culture, spheroids were dissociated using Cell Passaging Solution (Accutase; Pelo Biotech, Martinsried, Germany). After achieving a single cell suspension, fractions of ASCs and breast cancer cells were sorted utilizing Dynabeads^®^Epithelial Enrich (Thermo Fisher Scientific, Waltham, MA, USA) according to the manufacturer’s instructions. Subsequently, mRNA was isolated from both cell fractions as described for qRT-PCR. To exclude the impact of dissociation and sorting procedure on gene expression, respective controls were kept and analyzed as well. The dissociation and sorting procedure per se had no influence on the gene expression of target genes. For the evaluation of separation efficacy, ASCs were pre-stained with CellTracker™ CMFDA dye (Thermo Fisher) and the proportion of each cell type was counted in both fractions.

### 2.6. Quantification of Intracellular Triglyceride and DNA Content

Accumulation of intracellular lipids in differentiated ASCs was assessed as described in [28]. In brief, an analysis of triglyceride content was carried out using the triglyceride determination kit (Sigma-Aldrich). Cells were harvested in 0.5% aqueous Thesit solution (0.5% Thesit in H_2_O; Gepepharm, Hennef, Germany) and subsequently sonicated (Sonopuls; Bandelin electronic, Berlin, Germany). Triglyceride content was quantified according to the manufacturer’s instructions with a spectrofluorometer (Tecan GENios pro; Tecan, Crailsheim, Germany) at 570 nm. To quantify the DNA content of the samples, cells were suspended in phosphate/saline buffer (50 mM phosphate buffer, 2 mM Na_2_EDTA × 2 H_2_O, 2 M NaCl, pH 7.4; all purchased from Carl Roth, Karlsruhe, Germany) and sonicated. DNA-intercalating dye Hoechst 33258 (Polysciences, Warrington, PA, USA) was used to determine DNA content fluorometrically (Tecan GENios pro; Tecan) at an excitation wavelength of 365 nm and an emission wavelength of 458 nm. Salmon sperm DNA served as a standard. Intracellular TG contents were normalized to the total amount of DNA in the corresponding sample.

### 2.7. Quantitative Reverse Transcription Polymerase Chain Reaction (qRT-PCR)

For gene expression analysis, spheroids were harvested and subsequently homogenized in TRIzol^®^reagent (Life Technologies), followed by mRNA isolation according to the manufacturer’s instructions. cDNA synthesis from 1000 ng of total RNA was carried out using a High-Capacity cDNA Reverse Transcription Kit (Thermo Fisher Scientific). qRT-PCR was performed using self-designed primer pairs (Table 1). Mesa Green qPCR MasterMix Plus MeteorTaq polymerase (Eurogentec, Seraing, Belgium) was used for detection. The 2^−∆Δ Ct^ method was applied to determine the x-fold increase in mRNA expression levels for each gene [29], and the values obtained were further normalized to the respective monoculture values as indicated.

### 2.8. Detection of Secreted CCL5

Supernatants of the different culture conditions were collected after two days of culture (co- and monocultures) and stored at −20 °C until use. CCL5 concentrations were determined utilizing human CCL5/RANTES DuoSet ELISA (R&D Systems, Minneapolis, MN, USA) according to the manufacturer’s instructions. CCL5 levels were normalized to the total DNA contents of the corresponding samples.

### 2.9. Immunohistochemical Analyses

Immunohistochemical stainings were performed according to [28]. In brief, spheroids were fixed in 3.7% PFA (Carl Roth, Karlsruhe, Germany) at room temperature for 1 h, embedded in Tissue Tek O.C.T. (Sakura Finetek, Torrance, CA, USA), and dehydrated. Subsequently, samples were snap frozen in liquid nitrogen and sectioned using a cryostat (6 µm sections; CM 3050S Leica, Wetzlar, Germany). Antigens were retrieved by proteinase K digestion for 10 min at room temperature, followed by three washing steps with PBS. After blocking with 1% bovine serum albumin, sections were incubated with the primary antibody in 1% BSA in PBS. Perilipin-1 staining (1:400; PA5-72921, Thermo Fisher) was used to confirm adipogenic differentiation and the presence of lipid vacuoles in adipocyte-spheroids. To visualize tumor cells in co-culture spheroids, pan-cytokeratin AE1 /AE3 (1:100; ab27988, Abcam) co-staining was carried out. CCL5 receptors were stained using αCCR1 and αCCR5 antibodies (1:200; PA5-112058, Thermo Fisher Scientific and 1:200; ab7346, Abcam respectively). After overnight incubation at room temperature, samples were washed with PBS and incubated with secondary antibodies (goat anti-rabbit Alexa488-conjugated (ab15007,1:400, Abcam); donkey anti-mouse Cy3-conjugated (1:200; 715-165-150, Jackson ImmunoResearch, West Grove, PA, USA)) for 1 h at room temperature. After three final washing steps in PBS, sections were mounted with DAPI mounting medium Immunoselect (Dako, Hamburg, Germany). Images were acquired using a fluorescence microscope (Olympus BX51/DP71).

### 2.10. Conditioned Media and Migration Assays

To generate conditioned media for migration assays, cells were cultured in growth medium without FBS. After two days, the medium was aspirated, centrifuged at 1000× *g*, and stored in Protein LowBind^®^ tubes (Eppendorf, Hamburg, Germany) at −20 °C until use. For migration assays, 3.5 × 10^5^ cells/mL were cultured in removable 2-well culture inserts inside 3-well slides (both Ibidi, Gräfeling, Germany). After an adherence period of 24 h in the growth medium, cells were washed twice with serum-free medium and then cultured for 24 h in serum-free medium for FBS starvation. The 2-well chambers were removed and then washed with serum-free medium, and the experimental conditions were applied. For migration assays using MCF-7, 6 × 10^5^ cells/mL were seeded per chamber. After an adherence period of 24 h in growth medium, cells were washed twice with serum-free medium, and conditions were applied. CCR1-antagonist BX471 (Tocris Bioscience, Bristol, UK) was used at a concentration of 30 µM, while CCR5 antagonist TAK-779 (Merck, Darmstadt, Germany) was used at a concentration of 1 µM [30]. Gap closure was documented after 0 h and 48 h and the percentage of area covered was assessed using FastTrackAI (MetaVi Labs, Austin, TX, USA).

### 2.11. Statistics

Statistical analyses were performed using OriginPro 2021b. Quantitative data are shown as mean ± standard deviation. Experiments were conducted at least three times with *n* = 3 replicates, if not stated otherwise. One-way or two-way analyses of variance (ANOVA) were used to determine statistical significance, followed by multiple comparisons by Bonferroni’s post hoc test. Values of *p* < 0.05 were considered statistically significant.

## 3. Results

### 3.1. Generation and Characterization of Co-Spheroids Using ASC and Breast Cancer Cells

To mimic the direct interaction between ASCs and breast cancer cells that occurs in the TME in vivo in a 3D environment, 3D multicellular tumor spheroids consisting of ASCs and breast carcinoma cell lines (MDA-MB-231 or MCF-7) were engineered in agarose-based micromolds. 

**Figure 2:**
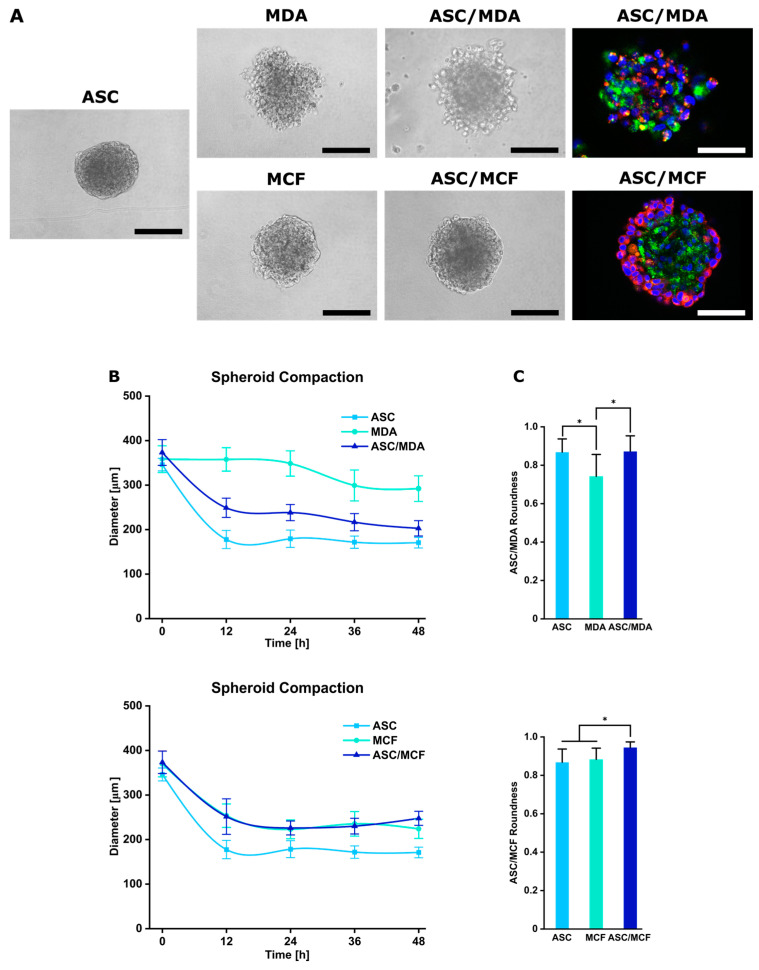
Characterization of mono- and co-culture spheroids of ASCs and MDA-MB-231 or MCF-7. (**A**) Mono- and co-culture spheroids of ASCs and MDA-MB-231 or MCF-7 after 48 h containing 1000 cells. Co-cultures of ASCs and breast cancer cell lines were pre-stained with PKH membrane dyes to visualize cell distribution (green: ASCs, red: cancer cells, blue: nuclei counterstained with DAPI). Bar equals 100 µm. (**B**) Spheroid compaction determined by measuring spheroid diameter of mono- and co-culture spheroids at different time points. (**C**) Shape of spheroids. Roundness was analyzed after 48 h using ImageJ, Version 1.53t. Data are presented as mean ± standard deviation (*n* = 80). Statistically significant differences (*p* < 0.05) are indicated by *.

This technique enabled the production of 256 spheroids per well and a total of 3072 spheroids per 12-well plate (Figure 1). Stromal cells and tumor cells were seeded in a 1:1 ratio and seeding concentration was adjusted to produce spheroids consisting of 1000 cells. Monoculture spheroids as controls were produced in the same manner using ASCs and MDA-MB-231 and MCF-7 cells. Spontaneous self-assembly of cells in the non-adhesive micromolds formed 3D multicellular spheroids with a single spheroid per well in the co-cultures as well as in the corresponding monocultures, as shown by phase contrast microscopy. While ASC and co-culture spheroids with either MDA-MB-231 and MCF-7 appeared round and compact, MDA-MB-231 monoculture spheroids were more irregularly shaped, but sufficiently cohesive (Figure 2A).

To visualize the presence and distribution of both cell types in the co-spheroids after the culture period of 48 h, ASCs and breast cancer cells were stained with green and red fluorescent PKH dyes prior to spheroid assembly. In ASC/MDA-MB-231 co-spheroids, both cell types were evenly distributed, whereas in ASC/MCF-7 co-cultures, cancer cells appeared predominantly located in the outer region of the spheroid around a core of ASCs (Figure 2A). To monitor the assembly process, images of the spheroids were taken at different time points and diameters of the cell aggregates were determined. In co-culture with ASCs, MDA-MB-231 cells readily formed aggregates within 12 h, which showed further compaction after 48 h, whereas monospheroids of MDA-MB-231 assembled more slowly and remained less compact after this time. Co-culture with MCF-7 cells formed compact spheroids within 12 h with no further reduction in spheroid diameter after 24 h, comparable to MCF-7 monospheroids. Spheroid sizes ranged from 170.75 µm (±11.95 µm) for ASC monospheroids to 292.1 µm (±28.83 µm) for MDA-MB-231 spheroids (Figure 2B). To further characterize the morphology of the generated spheroids, roundness as a parameter reflecting the circularity of the projected area was analyzed. It ranges from 0 to 1, with values close to 1 indicating high circularity [31]. As shown in Figure 2C, roundness values were high for ASC/MDA-MB-231 co-spheroids and comparable to ASC monospheroids, which reflects the well-rounded character and good cohesion of the co-spheroids. ASC, MCF-7, and ASC/MCF-7 spheroids displayed uniformly high roundness values.

Taken together, the applied technology enables the high-throughput generation of compact microtumors containing ASCs and breast carcinoma cell lines in a defined and reproducible 3D set-up that allows the study of direct cell–cell interactions between ASCs and breast cancer cells in this context.

### 3.2. CCL5 Expression Is Up-Regulated in 3D ASC/MDA-MB-231 Co-Spheroids

**Figure 3:**
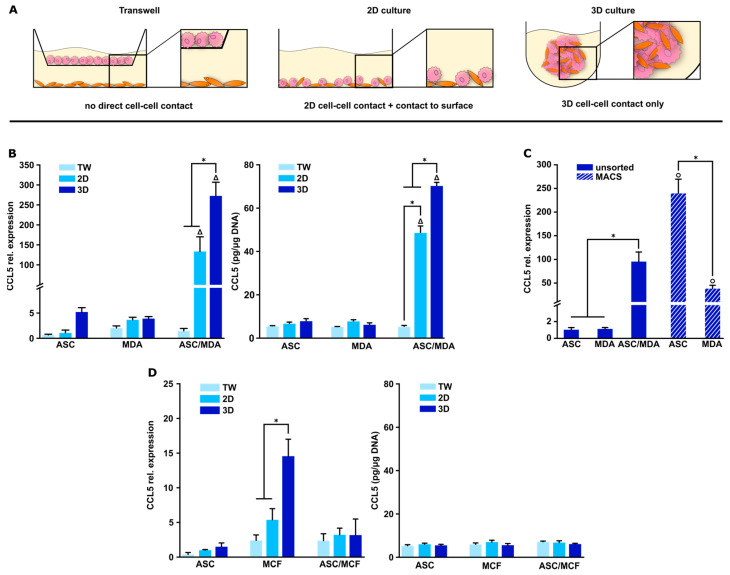
CCL5 expression in mono- and co-culture spheroids of ASCs and MDA-MB-231 or MCF-7 compared to indirect and direct 2D cultures. (**A**) Schematic illustration of analyzed co-culture systems. Transwell culture allows for indirect crosstalk of cells by paracrine signaling. Direct 2D culture provides direct contact between cells, but also relies on adhesion to plastic surfaces. Three-dimensional spheroid co-culture facilitates close cell–cell interaction in a tissue-like environment without adhesion of cells to artificial surfaces. (**B**) CCL5 gene expression (**left**) and protein secretion (**right**) in mono- and co-cultures of ASCs and MDA-MB-231 in varying culture systems after 48 h. Gene expression was assessed using qRT-PCR and was normalized to the housekeeping gene EF1α; obtained values were further normalized to standard 2D ASC monoculture. CCL5 protein secretion was determined by ELISA in culture supernatants and obtained values were normalized to the DNA contents of the respective samples. Data are presented as mean ± standard deviation (*n* = 3). * indicates statistically significant differences (*p* < 0.05) between culture systems; Δ indicates statistically significant differences (*p* < 0.05) to corresponding monocultures. (**C**) CCL5 gene expression after MACS sorting of 3D co-culture spheroids of ASCs and MDA-MB-231 cultured for 48 h. Three-dimensional mono- and co-spheroids were used as controls. Data are presented as mean ± standard deviation (*n* = 3). Gene expression was assessed using qRT-PCR and was normalized to the housekeeping gene EF1α; obtained values were further normalized to 3D ASC monoculture as MACS indicated CCL5 mostly originating from ASCs. * indicates statistically significant differences (*p* < 0.05); o indicates statistically significant differences (*p* < 0.05) to corresponding monocultures. (**D**) CCL5 gene expression (**left**) and protein secretion (**right**) in mono- and co-cultures of ASCs and MCF-7 in varying culture systems after 48 h. Gene expression was normalized to the housekeeping gene EF1α; obtained values were further normalized to standard 2D ASC monoculture. CCL5 protein secretion was determined by ELISA using culture supernatants and obtained values were normalized to DNA contents of respective samples. Data are presented as mean ± standard deviation (*n* = 3). * indicates statistically significant differences (*p* < 0.05) between culture systems.

CCL5 expression has been reported to be stimulated by the interaction of ASCs and breast cancer cells. However, there are divergent results regarding the necessity of direct cell–cell contacts for the expression of CCL5 in this context. To date, such analyses have been only carried out in direct and indirect 2D cultures [22,23]. In indirect 2D co-cultures using transwells, each cell type is located in its own compartment and only indirect cell–cell contact is possible through soluble factors. In 2D direct co-culture, cell–cell interactions are limited, and cells additionally may be influenced by the contact with the plastic surface, whereas in the spheroid model, cells are in close contact with each other in a 3D architecture, as illustrated in Figure 3A. Therefore, to study CCL5 expression in this more in vivo-like microenvironment, we used the newly established 3D co-culture spheroids (ASC/MDA-MB-231 and ASC/MCF-7). To evaluate the significance of direct cell–cell contacts and the dimensionality of the culture system in this context, we compared the expression in the 3D co-spheroids to conventional indirect (transwells) and direct 2D co-cultures of ASCs and breast cancer cells. The respective monocultures served as controls. For gene expression studies, uniform expression levels of the used housekeeping gene EF1alpha were confirmed in the different cell types and culture systems. As shown in Figure 3B, CCL5 gene expression was highly up-regulated in direct co-culture with the triple-negative cell line MDA-MB-231, while expression in indirect co-culture was comparable to the low expression level in the respective monocultures. In 3D spheroids, the expression was further significantly increased (272-fold) compared to direct 2D culture (133-fold, both relative to ASC monoculture). The gene expression pattern was mirrored in protein expression levels. CCL5 secretion was markedly increased in direct ASC/MDA-MB-231 co-culture with further increase in 3D spheroids compared to direct 2D co-culture, whereas CCL5 concentration remained at a base level in monocultures and in indirect 2D co-culture (Figure 3B). Thus, CCL5 expression was strictly dependent on direct cell–cell contacts between ASCs and MDA-MB-231 cells and was significantly elevated in 3D co-spheroids compared to conventional 2D co-culture. To be able to determine the origin of the CCL5 in the ASC/MDA-MB-231 co-culture spheroids, magnetic-activated cell sorting (MACS) technology was established. For this purpose, spheroids were dissociated and separated into distinct cell fractions of ASCs and tumor cells, resulting in highly enriched fractions of ASC and MDA-MB-231 cells (Figure S1). Gene expression analysis revealed a 239-fold increase in the ASC fraction upon direct contact with the tumor cells compared to the ASCs in spheroid monoculture, whereas MDA-MB-231, in contrast, showed a markedly lower expression level (Figure 3C). Therefore, the highly elevated CCL5 expression in direct 3D ASC/MDA-MB-231 co-spheroids can be mainly attributed to the ASC fraction. In co-culture with less aggressive MCF-7 tumor cells, no up-regulation was evident in 3D co-cultures compared to indirect and direct 2D co-cultures as well as the respective monocultures; solely in the MCF-7 monocultures, an increase was observed in the 3D spheroids compared to the 2D cultures. Analysis of CCL5 protein secretion in ASC/MCF-7 co-culture revealed only base levels of released protein and no up-regulation upon any form of co-culture (Figure 3D).

These findings highlight the importance of direct cell–cell contacts of ASCs and triple-negative breast cancer cells for CCL5 expression and the relevance of our 3D spheroid model for studying such cell–cell interactions.

### 3.3. Expression of CCR1 Receptor Is Specifically Increased in 3D ASC/MDA-MB-231 Co-Spheroids

**Figure 4:**
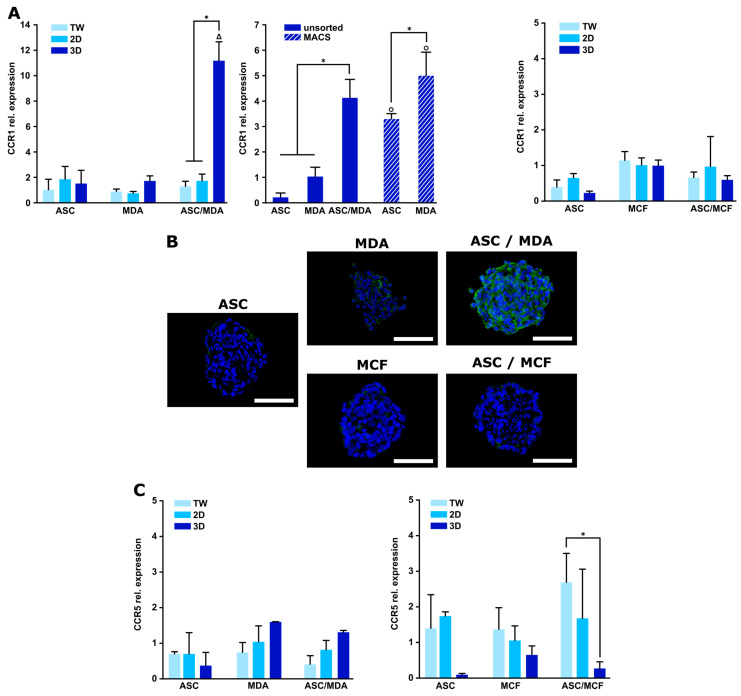
Expression of CCL5 receptors CCR1 and CCR5 in mono- and co-culture spheroids of ASCs and MDA-MB-231 or MCF-7 compared to indirect and direct 2D cultures. (**A**, **left**) CCR1 gene expression in mono- and co-cultures of ASCs and MDA-MB-231 in varying culture systems and after MACS sorting of 3D co-spheroids after 48 h. Gene expression was assessed using qRT-PCR and was normalized to the housekeeping gene EF1α; obtained values were further normalized to standard 2D MDA-MB-231 monoculture or to 3D MDA-MB-231 monoculture after MACS sorting. Data are presented as mean ± standard deviation (*n* = 3). * indicates statistically significant differences (*p* < 0.05) between culture systems; Δ indicates statistically significant differences (*p* < 0.05) to corresponding monocultures, o indicates statistically significant differences (*p* < 0.05) to corresponding monocultures after MACS sorting. (**A**, **right**) CCR1 gene expression in mono- and co-cultures of ASCs and MCF-7 in varying culture systems. Gene expression was assessed using qRT-PCR and was normalized to the housekeeping gene EF1α; obtained values were further normalized to standard 2D MCF-7 monoculture. Data are presented as mean ± standard deviation (*n* = 3). (**B**) CCR1 protein expression in mono- and co-cultures of ASCs and MDA-MB-231 or MCF-7 cells. Immunohistochemical staining for CCR1 was conducted. CCR1 was stained green and nuclei blue with DAPI. Representative images are shown. Scale bar represents 100 µm. (**C, left**) CCR5 gene expression in mono- and co-cultures of ASCs and MDA-MB-231 in varying culture systems after 48 h. Gene expression was assessed using qRT-PCR and was normalized to the housekeeping gene EF1α; obtained values were further normalized to standard 2D MDA-MB-231 monoculture. Data are presented as mean ± standard deviation (*n* = 3). (**C, right**) CCR5 gene expression in mono- and co-cultures of ASCs and MCF-7 in varying culture systems after 48 h. Gene expression was assessed using qRT-PCR and was normalized to the housekeeping gene EF1α; obtained values were further normalized to standard 2D MCF-7 monoculture. Data are presented as mean ± standard deviation (*n* = 3). * indicates statistically significant differences (*p* < 0.05) between culture systems.

To complementarily characterize the expression of CCL5 receptors and to gain further insight into the functionality of CCL5 in this model, expression levels of the main CCL5 receptors CCR1 and CCR5 were analyzed in the 3D co-culture spheroids and compared to indirect and direct 2D co-cultures. As shown in Figure 4A, CCR1 was specifically up-regulated upon direct contact in 3D co-cultures of ASCs and MDA-MB-231 cells, whereas comparably low expression levels were observed in indirect and direct 2D co-culture as well as in the monocultures under all conditions. This is in accordance with immunohistochemical staining for CCR1 protein expression, which revealed distinct staining in ASC/MDA-MB-231 co-spheroids, whereas only a weak signal was observed in the corresponding monocultures (Figure 4B).

Differential expression analysis of CCR1 in MACS-sorted cell fractions of ASC and MDA-MB-231 after 3D co-culture revealed that CCR1 expression was up-regulated to a significantly higher extent in the cancer cell fraction in comparison to the ASC fraction. The reciprocal expression of CCL5 predominantly in ASCs (Figure 3C) and CCR1 largely in the tumor cells (Figure 4A) may indicate a specific chemokine receptor interaction in the 3D co-spheroids. In contrast, the expression of CCR5, another main receptor of CCL5, showed no significant up-regulation in the co-cultures compared to the respective monocultures in gene (Figure 4C) and protein expression (Figure S2). To complete the analysis of CCL5 receptors, CCR3 expression was also examined, and again, no distinct up-regulation was detected in co-cultures compared to the monocultures (Figure S3A).

In 3D ASC/MCF-7 co-spheroids, CCR1 expression was not significantly modified compared to direct or indirect 2D co-cultures or monocultures in terms of both gene and protein expression (Figure 4A,B). CCR5 and CCR3 also showed no regulation of expression in the co-cultures (2D or 3D) compared with the respective monocultures (Figure 4C and Figure S3B). Thus, consistent with the CCL5 expression pattern in these co-cultures, direct 3D cell contact between ASCs and MCF-7 cells did not trigger CCL5 receptor expression.

### 3.4. Blocking of CCR1 Inhibits Migration of Triple-Negative Cancer Cells Mediated by 3D ASC/MDA-MB-231 Co-Culture

**Figure 5:**
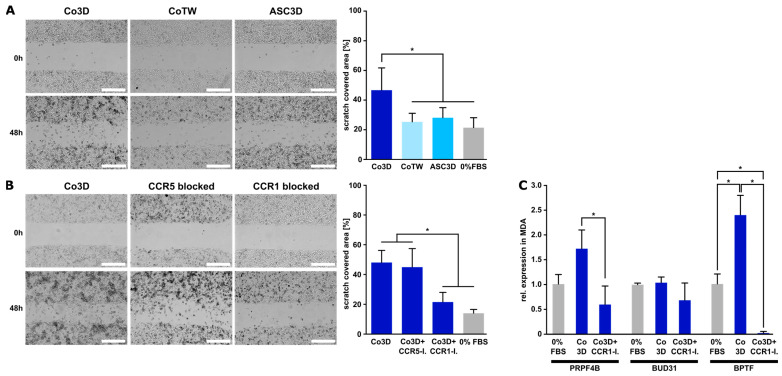
Impact of conditioned media from different culture forms and CCL5 receptor blocking on MDA-MB-231 migration. (**A**) Migration (scratch) assay of MDA-MB-231 in conditioned media from 3D ASC/MDA-MB231 co-spheroids (Co3D), indirect transwell co-culture (CoTW), and ASC monospheroids (ASC 3D). Growth medium without FBS served as further control (0% FBS). Representative micrographs were taken at 0 h and 48 h for illustration. Bar equals 500 µm. Images acquired after 48 h were analyzed using FastTrackAI™. Data are presented as mean ± standard deviation (*n* = 12). * indicates statistically significant differences (*p* < 0.05). (**B**) Specific blocking of CCL5 receptors CCR1 and CCR5. Specific CCR1 (BX471) and CCR5 (TAK-779) antagonists were added to the conditioned medium of 3D co-spheroids (Co3D+CCR5-I, Co3D+CCR1-I) and migration was assessed in comparison to untreated conditioned medium from 3D co-spheroids (Co3D). Growth medium without FBS served as further control (0% FBS). Images were acquired and analyzed as described above. Bar equals 500 µm. Data are presented as mean ± standard deviation (*n* = 12). * indicates statistically significant differences (*p* < 0.05). (**C**) Gene expression of migration-related markers (PRPF4B, BUD31, BPTF) in MDA-MB-231 cells upon stimulation with CM from ASC/MDA-MB-231 co-spheroids and addition of CCR1 inhibitor BX471. Gene expression was assessed using qRT-PCR and was normalized to the housekeeping gene EF1α; obtained values were further normalized to 0% FBS control. Data are presented as mean ± standard deviation (*n* = 3). * indicates statistically significant differences (*p* < 0.05) between conditions.

An intrinsic feature of triple-negative breast cancer cells is their migration behavior. To investigate the functional relevance of the proposed CCL5/CCR1 axis for the migratory ability of triple-negative breast cancer cells, migration assays were performed with MDA-MB-231 cells using a standard scratch assay. Conditioned media (CM) from 3D co-culture spheroids and, for comparison, CM from indirect co-culture (transwell) and ASC monoculture were used in the assay and scratch closure was observed. As shown in Figure 5A, CM from the 3D co-spheroids significantly enhanced the migration of MDA-MB-231 cells, whereas CM from ASC monoculture spheroids as well as from indirect co-culture had no effect on migration. This indicated the relevance of direct cell–cell interaction of these cell types for the migratory ability of the cancer cells. The functionality of CCR1 was further investigated by adding a specific CCR1 receptor antagonist (BX471) to CM of 3D co-cultures. Blocking CCR1 distinctly reduced the migration-enhancing effect of the 3D co-culture, whereas the addition of a specific CCR5 inhibitor (TAK-779) did not affect the migration behavior of MDA-MB-231 cells (Figure 5B). In contrast, migration of MCF-7 cells was not affected by CM from ASC/MCF-7 co-spheroids or the addition of CCR1 or CCR5 inhibitor (Figure S4).

We further examined the expression of migration-related marker genes in MDA-MB-231 cells (PRPF4B, BUD31, BPTF) upon stimulation with co-spheroid CM or after the addition of the CCR1 inhibitor. Gene expression of two of these migration-related markers (PRPF4B and BPTF) was significantly up-regulated upon stimulation with CM from the co-spheroids, while the addition of the CCR1 inhibitor BX471 caused a marked reduction in the CM-stimulated expression level. BUD31 expression was not affected by CM stimulation or BX471 addition (Figure 5C). Altogether, these data strongly indicate a possible role of CCR1 in the migration ability of triple-negative breast cancer cells in an adipose microenvironment.

### 3.5. Generation and Characterization of Co-Spheroids Using Adipocytes and Breast Cancer Cells

**Figure 6:**
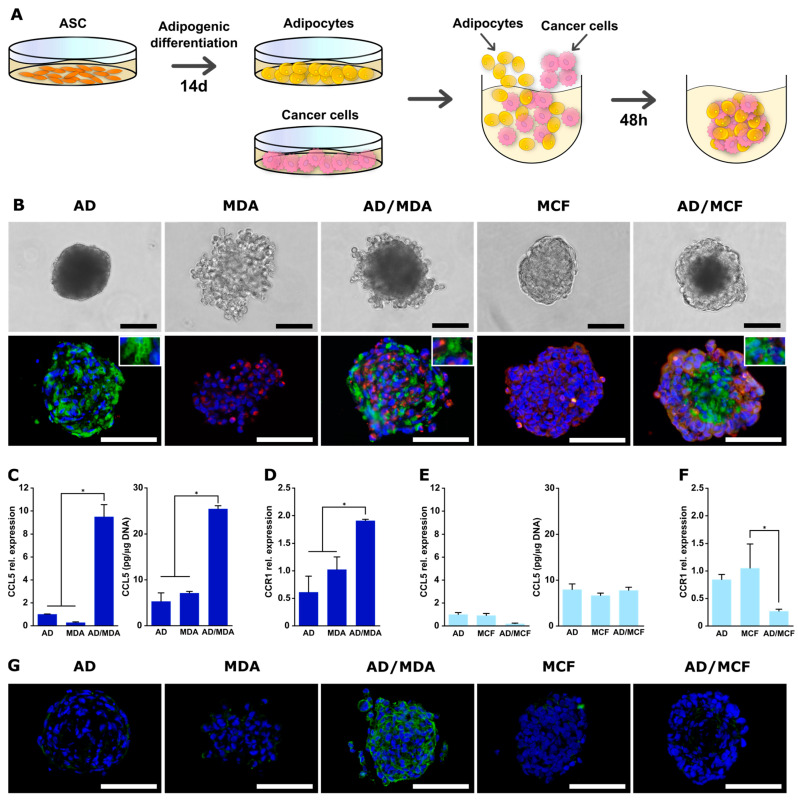
Characterization and CCL5/CCR1 expression of mono- and co-culture spheroids of adipocytes and MDA-MB-231 or MCF-7. (**A**) Schematic illustration of spheroid formation. ASCs were adipogenically differentiated in 2D culture for 14 days, followed by detachment and co-seeding with tumor cells in agarose micromolds. Cells were permitted to form spheroids over 48 h. (**B**) Mono- and co-culture spheroids of adipocytes (AD) and MDA-MB-231 or MCF-7 after 48 h. Mono- and co-spheroids were immunohistochemically stained for perilipin-1 (green: adipocytes) and pan-cytokeratin (red: cancer cells). Nuclei were stained with DAPI (blue). Bar equals 100 µm. (**C**) CCL5 gene expression (**left**) and protein secretion (**right**) in mono- and co-cultures of adipocytes and MDA-MB-231 in 3D spheroids after 48 h. Gene expression was assessed using qRT-PCR and was normalized to the housekeeping gene EF1α; obtained values were further normalized to 3D adipocyte monoculture. CCL5 protein secretion was determined by ELISA in supernatants and obtained values were normalized to the DNA contents of the respective samples. (**D**) CCR1 gene expression in mono- and co-cultures of adipocytes and MDA-MB-231 in 3D spheroids after 48 h. Gene expression was assessed using qRT-PCR and was normalized to the housekeeping gene EF1α; obtained values were further normalized to 3D MDA-MB-231 monoculture. (**E**) CCL5 gene expression (**left**) and protein secretion (**right**) in mono- and co-cultures of adipocytes and MCF-7 in 3D spheroids after 48 h. Gene expression was assessed using qRT-PCR and was normalized to the housekeeping gene EF1α; obtained values were further normalized to 3D adipocyte monoculture. CCL5 protein secretion was determined by ELISA in supernatants and obtained values were normalized to the DNA contents of the respective samples. (**F**) CCR1 gene expression in mono- and co-cultures of adipocytes and MCF-7 in 3D spheroids after 48 h. Gene expression was assessed using qRT-PCR and was normalized to the housekeeping gene EF1α; obtained values were further normalized to 3D MCF-7 monoculture. All data are presented as mean ± standard deviation (*n* = 3). * indicates statistically significant differences (*p* < 0.05) between culture systems. (**G**) CCR1 protein expression in mono- and co-cultures of adipocytes and MDA-MB-231 or MCF-7 cells. Immunohistochemical staining for CCR1 was conducted. CCR1 was stained green and nuclei blue with DAPI. Representative images are shown. Scale bar represents 100 µm.

To be able to also study the direct interaction between adipocytes and tumor cells, we aimed to establish co-spheroids using adipogenically differentiated ASCs. Adipogenic differentiation of ASCs was performed using a common hormonal cocktail for 14 days. After this differentiation phase, the cells exhibited a lipid-rich phenotype, as shown by lipid droplet staining and quantification of the lipid content. The expression of key adipogenic marker genes for early (PPARγ, C/EBPα) and late (FABP4) adipogenesis were distinctly up-regulated, confirming the phenotype of differentiated adipocytes (Figure S5). Three-dimensional spheroids with these adipocytes were generated in the same manner as described for ASC spheroids (Figure 6A). Adipocytes alone or in combination with cancer cells were seeded in the non-adhesive agarose micromolds and readily formed spheroidal aggregates within 48 h. Adipocyte monospheroids as well as co-spheroids with MCF-7 displayed a round, compact structure, whereas adipocyte/MDA-MB-231 spheroids were slightly frayed. The presence of lipid droplet-containing adipocytes as well as breast cancer cells in co-spheroids was confirmed by co-staining of perilipin-1 (adipocytes) and pan-cytokeratin (tumor cells) (Figure 6B). Adipocyte/MDA-MB-231 co-spheroids exhibited an apparently random distribution of both cell types. In adipocyte/MCF-7 co-cultures, cancer cells were present mainly in the outer regions of the spheroids, as previously observed for ASC/MCF-7 co-spheroids (Figure 2A). The assembly and compaction process of adipocyte-containing spheroids was slightly retarded compared to ASCs (Figure 2B and Figure S6). Final spheroid size ranged from 196.1 µm (±17.92 µm) for adipocytes alone to 261.48 µm (±18.02 µm) for adipocyte/MDA-MB-231 co-spheroids (Figure S6). Roundness for adipocyte mono- and co-spheroids also displayed high values comparable to those for spheroids with undifferentiated ASCs (Figure S6). Thus, our approach enabled the production of 3D co-spheroids containing adipocytes and tumor cells in a 3D environment with close cell–cell contacts.

As a proof-of-concept, we determined whether CCL5/CCR1 expression is also stimulated in adipocyte/MDA-MB-231 co-spheroids. As shown in Figure 6C, expression of CCL5 in these 3D co-spheroids was significantly up-regulated at both the gene and protein expression levels compared to the respective monocultures, albeit at lower levels than in the ASC-containing co-culture. In parallel, CCR1 expression was also significantly increased in the co-spheroids in comparison to the monospheroids at the gene and protein expression level (Figure 6D,G). Co-culture with MCF-7 did not result in the up-regulation of CCL5 gene expression or protein secretion, nor of CCR1 expression (Figure 6E–G).

Thus, also using the established co-spheroids composed of adipocytes and breast cancer cells, the expression of tumor-promoting factors (CCL5/CCR1) depending on the type of breast cancer cell could be demonstrated in a 3D environment.

## 4. Discussion

Breast carcinoma develops in close proximity to mammary adipose tissue and there is growing evidence that interactions with the local adipose environment drive tumor progression and metastasis [32,33]. The influence of fat-resident cells such as adipocytes and ASCs on breast carcinoma cells and vice versa has been extensively studied in vitro, mostly by using conditioned media or by 2D co-culture in transwell systems, as these techniques are easily feasible and well reproducible [34]. However, these approaches, while useful, allow only paracrine signaling between cells to be analyzed. Histological sections of breast carcinoma display adipose cells and tumor cells in close physical contact at the invasive tumor front [35,36]. The relevance of direct cell contacts in the tumor–stroma as a tumor-promoting effect in addition to paracrine signaling has been emphasized in breast cancer and other tumors [37,38]. Thus, advanced co-culture models mimicking the juxtacrine interaction between breast carcinoma cells and fat-resident cells such as adipocytes or ASCs in a 3D environment that recapitulates close cell–cell adhesion in tissues would extend the understanding of this complex bi-directional crosstalk.

For this purpose, we developed a 3D co-culture spheroid model consisting of breast carcinoma cells (MDA-MB-231 and MCF-7) and ASCs or adipocytes that meets these requirements.

First, co-spheroids of ASCs and breast cancer cells were generated in agarose-based micromolds in a high-throughput manner and with high reproducibility concerning parameters such as size and roundness. Spheroids generally presented a round-type morphology, which is indicative of strong cell–cell adhesion [39]. In co-spheroids of ASCs and MDA-MB-231 cells, both cell types were evenly distributed, whereas spheroids with MCF-7 cells displayed a heterogeneous distribution within the spheroids, with tumor cells surrounding a central core of ASC. Such defined co-culture models that reflect the direct crosstalk between ASCs and breast cancer cells in a three-dimensional set-up are rare. Bae et al. used ASC/MDA-MB-231 spheroids to investigate ECM remodeling and drug efficacy [40].

To evaluate our 3D co-spheroid model and to demonstrate the impact of the aforementioned direct heterotypic cellular contacts between breast cancer cells and ASCs, we specifically investigated the expression of CCL5 in the 3D co-spheroids in comparison to standard direct and indirect (transwell) 2D co-cultures. CCL5 is a major metastasis-promoting inflammatory chemokine and is known to drive pro-oncogenic tumor–stroma interactions [41,42]. In biopsies from breast cancer patients, the local CCL5 protein expression was observed to be elevated in invasive breast carcinomas compared to in situ ductal tumors or benign lesions [43,44], and CCL5 expression was immunolocalized in biopsies specifically representing the tumor–stroma border [23]. Several in vitro studies reported increased expression of CCL5 in co-cultures of breast cancer cells (mainly triple-negative MDA-MB-231 cells) and mesenchymal stromal cells such as ASCs, but also bone marrow-derived mesenchymal stromal cells (BMSCs), but these studies have so far only been conducted in standard 2D co-cultures [9,17,18,22,23]. There is evidence that the expression of CCL5 in this context requires direct cell–cell contact [17,18,23]. However, results have been inconsistent and this may be due to the use of cells with different origins (murine vs. human platform, ASCs vs. BMSCs) or different culture conditions. In particular, two studies specifically using ASCs as co-cells reported divergent results regarding the contact dependence of CCL5 expression [22,23].

Thus, we investigated CCL5 expression in the newly established ASC/MDA-MB-231 co-spheroids. We were able to demonstrate strong up-regulation of this chemokine in the 3D co-spheroids on the gene and protein expression level compared to direct 2D co-cultures. No expression could be detected in indirect transwell cultures. These results emphasize the relevance of close cell–cell contacts for CCL5 expression, which are much more pronounced in the co-spheroids due to their 3D architecture. Through dissociation and subsequent sorting of both cell types using MACS technology, ASCs were determined as the main source of CCL5 expression in the co-culture. This is in accordance with previous reports demonstrating that the main producers of tumor-associated CCL5 are not the tumor cells themselves, but the local mesenchymal stromal cell population [17,22,45]. Up-regulation of CCL5 secretion was not evident when MCF-7 cells were used as co-cells in the co-culture instead of MDA-MB-231.

Chemokines exert their action through their cognate chemokine C-C receptors (CCRs), which mostly belong to the G-protein-coupled receptor (GPCR) superfamily [41]. Several chemokines can bind to more than one receptor, although the exact profile of the chemokine receptor expression on an individual cell depends, in part, on microenvironmental factors such as chemokine concentration or inflammatory milieu [46]. CCL5 acts through three GPCRs, termed CCR1, CCR3, and CCR5. As we observed a highly elevated CCL5 secretion in our 3D ASC/breast cancer model, we wanted to pursue this further and characterize the expression of the corresponding receptors. A marked up-regulation of CCR1 expression was evident only in 3D co-spheroids of ASCs and MDA-MB-231 at both gene and protein expression levels, while no increase was observed in indirect or direct 2D co-culture. The close contact between ASCs and triple-negative breast carcinoma cells in the 3D environment appeared to be a distinct stimulus that specifically up-regulated CCR1 expression. In contrast, CCR3 and CCR5 expression was not significantly altered upon 3D co-culture of both cell types. Differential expression analysis in the MACS-sorted cells revealed a significantly higher expression of CCR1 in the tumor cells compared to the ASCs. CCR1 expression in MCF-7 cells was not enhanced upon 3D co-culture with ASCs. Thus, the reciprocal expression pattern of CCL5 and specifically CCR1 suggests a predominant chemokine/receptor interaction between CCL5-producing ASCs and CCR1-expressing basal breast cancer cells in the 3D co-spheroids. CCR1 has recently been implicated in tumor invasion and metastasis in various cancers, such as prostate cancer, colon cancer, and hepatocellular carcinoma [47,48,49]. A study conducted by Shin et al. suggested a function of CCR1 in the invasion of breast cancer cells as they inhibited the invasive capability of MDA-MB-231 cells through CCR1 silencing and demonstrated stronger CCR1 immunoreactivity in biopsies from invasive ductal carcinoma than in adjacent normal mammary tissue [50]. Other studies have reported CCR5 as the main receptor for CCL5 in the interaction between breast cancer cells and stromal cells; however, these studies have exclusively been conducted using 2D culture models and BMSCs [17,41,45,51]. To evaluate the functionality of the proposed CCL5/CCR1 axis, we conducted migration assays with MDA-MB-231 cells, exposing them to conditioned media of the 3D co-spheroids. A distinct migration-promoting effect could be observed under this condition compared to conditioned media from indirect co-cultures or monocultures, which was markedly reduced upon the addition of a specific CCR1 inhibitor. Blockage of CCR5 did not exert any effect. The up-regulation of migration-related markers (PRPF4B, BPTF) upon stimulation with CM from the co-spheroids, which was abrogated by the addition of the CCR1 inhibitor, was consistent with the migration behavior under these conditions. PRPF4B and BPTF act as transcriptional modulators and are described as part of the complex gene network that defines the migratory program of triple-negative breast cancer cells [52]. This strongly suggests that CCR1 plays a functional role in the migration behavior of basal breast cancer cells in an environment enabling close cell–cell contacts between ASCs and tumor cells, as found in invasive carcinomas in the mammary fat pad [32].

Adipocytes constitute another important cellular component of the mammary fat pad and represent the most abundant cell type surrounding breast cancer cells. Accumulating evidence suggests that adipocytes are active players in the tumor microenvironment by promoting the progression of cancer cells [32,33,53]. Therefore, we have further developed the 3D co-spheroid model using adipocytes as co-cells in order to mimic the direct cell–cell contact between adipocytes and breast cancer cells. To our knowledge, this is the first time that differentiated adipocytes have been integrated into a heterotypic breast cancer spheroid model to allow direct 3D cell–cell interactions between adipocytes and breast cancer cells to be studied. The assembly of the co-spheroids containing adipocytes was slightly retarded, but also yielded defined, well-rounded aggregates. Immunohistochemical staining of lipid droplets demonstrated the successful incorporation of adipocytes in the co-spheroids. Other 3D adipocyte/breast cancer models have been published, but these usually had the adipocytes or tumor cells embedded in hydrogel and co-cells seeded on top, so that no direct cell-to-cell contact between adipocytes and tumor cells could take place [28,54,55,56,57]. This is in contrast to our co-spheroid model, which, due to its 3D architecture without scaffolding materials, ensures a multitude of cell–cell contacts between these two cell types. As a proof-of-concept, the adipocyte/breast cancer spheroid model was used to analyze the expression of CCL5 and CCR1 complementary to the ASC co-spheroid model. CCL5 and CCR1 expression were significantly up-regulated in the co-spheroids with MDA-MB-231 compared to the respective monocultures, albeit to a lesser extent than in ASC/MDA-MB-231 co-spheroids. In adipocyte/MCF-7 co-spheroids, no increase in the expression of both markers was detected. A previous study has also demonstrated increased expression of CCL5 in direct 2D co-cultures of adipogenically differentiated ASCs and MDA-MB-231, but has not examined CCL5 receptor expression in this context [58].

Utilizing our newly developed co-spheroid model, we were able to demonstrate in this proof-of-concept study, using the CCL5/CCR1 axis as an example, the relevance of such 3D models for deciphering potentially new markers resulting from the direct interaction between breast cancer cells and adipose stroma cells that may not be detected in standard 2D culture. Future investigations, e.g., using gene silencing, may further validate the impact of the CCL5/CCR1 interaction on breast cancer progression in a 3D adipose microenvironment. The inclusion of primary patient-derived cells can further increase the relevance of the proposed 3D model and could pave the way toward more personalized diagnosis and therapy options that specifically target individually expressed or up-regulated markers in single patients. In addition, the high-throughput generation of these microtumors with defined composition and geometry may allow more accurate profiling of contact-induced markers in an in vivo-like 3D environment through transcriptome or proteome analyses, potentially revealing new targets for diagnostic and therapeutic options in breast cancer. Knowledge of such markers or ligand–receptor interactions could help to offer new targets for clinical therapeutic approaches. For example, they could be used for imaging tumor cell invasion into host tissue followed by endoradiotherapy using the same target molecule, a strategy that has already been implemented clinically for the receptor CXCR4, with CXCR4-directed molecular imaging and radioligand therapy [7,59].

## 5. Conclusions

With the established advanced 3D co-spheroid model, we provide a high-throughput and reproducible 3D platform to mimic the direct interaction of breast cancer cells and adipose tissue-inherent cells such as ASCs and adipocytes in a tissue-like context. A markedly up-regulated expression of the chemokine CCL5 and its receptor CCR1 was revealed in the 3D co-spheroid model, which was shown to be strongly contact-dependent. CCR1 functionality for migration of basal breast cancer cells was proven. Thus, the context-dependent expression of the CCL5/CCR1 axis shown in the 3D co-spheroids may act as a tumor-promoting factor when tumor cells and mammary fat cells come into close contact in a 3D environment, as is the case with the infiltration of breast cancer cells in adjacent adipose tissue. These findings highlight the potential of our 3D model as a contribution to deciphering the intricate interaction between adipose tissue and breast cancer in a more in vivo-like context to potentially identify novel markers that could provide therapeutic targets in the treatment of aggressive breast cancer.

## Figures and Tables

**Table 1:** Primer sequences for qRT-PCR.

Primer	Sequence
EF1α forward	5′-CCCCGACACAGTAGCATTTG-3′
EF1α reverse	5′-TGACTTTCCATCCCTTGAACC-3′
CCL5 forward	5′-CTGCTGCTTTGCCTACATTG-3′
CCL5 reverse	5′-TGTACTCCCGAACCCATTTC-3′
CCR1 forward	5′-CCAATGGGAATTCACTCACC-3′
CCR1 reverse	5′-GAGCCTGAAACAGCTTCCAC-3′
CCR3 forward	5′-GTGTTCACTGTGGGCCTCTT-3′
CCR3 reverse	5′-GTGACGAGGAAGAGCAGGTC-3′
CCR5 forward	5′-CTGCCTCCGCTCTACTCACT-3′
CCR5 reverse	5′-GCTCTTCAGCCTTTTGCAGT-3′
PPARγ forward	5′-TTCAGAAATGCCTTGCAGTG-3′
PPARγ reverse	5′-CCAACAGCTTCTCCTTCTCG-3′
C/EBPα forward	5′-TGGACAAGAACAGCAACGAG-3′
C/EBPα reverse	5′-TTGTCACTGGTCAGCTCCAG-3′
FABP4 forward	5′-CATACTGGGCCAGGAATTTG-3′
FABP4 reverse	5′-TACCAGGACACCCCCATCTA-3′
BPTF forward	5′-TGAAGAAATCCACCGACACA-3′
BPTF reverse	5′-CTCCCTTTTTGGCTCTTATGG-3′
BUD31 forward	5′-TTGATTGAGCCAACACTGGA-3′
BUD31 reverse	5′-ATGTAG CGGGTTTTCTGGTG-3′
PRPF4B forward	5′-ACGACGAGAACCAGAGAGGA-3′
PRPF4B reverse	5′-GGCATCTTTTGATCTTTCACG-3′

## Data Availability

Data are contained within the article.

## Supplementary Materials

The following supporting information can be downloaded at: https://www.mdpi.com/article/10.3390/cancers15133501/s1, Supplementary Figure S1: Evaluation of MACS; Supplementary Figure S2: Protein expression of CCR5 in mono- and co-culture spheroids of ASC und MDA-MB-231 and MCF-7 after 48 h; Supplementary Figure S3: CCR3 gene expression in mono- and co-cultures of ASCs and MDA-MB-231 or MCF-7 in varying culture systems; Supplementary Figure S4: Impact of CCL5 receptor blocking on MCF-7 migration; Supplementary Figure S5: Characterization of differentiated adipocytes; Supplementary Figure S6: Characterization of spheroids containing adipocytes and MDA-MB-231 or MCF-7.
