# Supplementary material for: Three-Dimensional Breast Cancer Model to Investigate CCL5/CCR1 Expression Mediated by Direct Contact between Breast Cancer Cells and Adipose-Derived Stromal Cells or Adipocytes
Source: Cancers (Basel). 2023 Jul 5;15(13):3501. doi: 10.3390/cancers15133501 (PMC10341373; doi:10.3390/cancers15133501)
Supplement: Supplementary file 1 [file cancers-15-03501-s001.zip › cancers-2429428-supplementary.pdf]

cancers

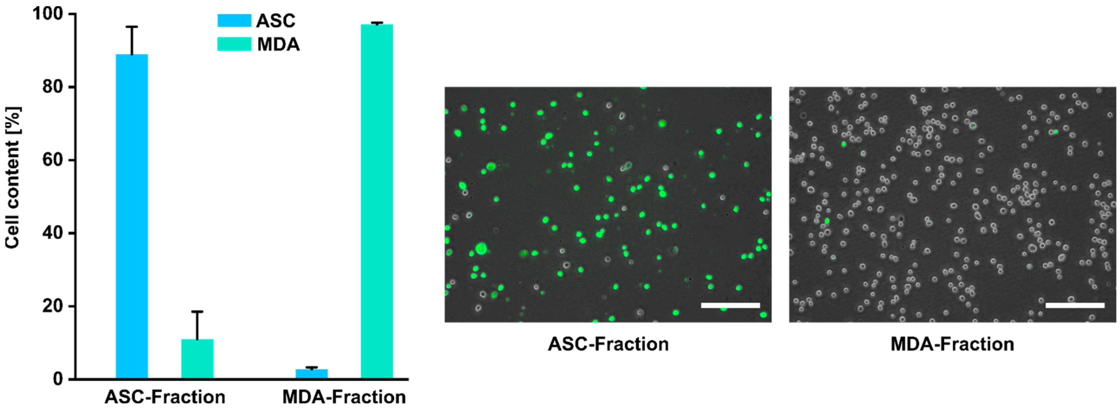

Supplementary Figure S1: Evaluation of MACS. ASC were prestained with CellTracker™ CMFDA dye prior to spheroid formation. Spheroids contained 1000 cells and were cultured for 48h. After dissociation and separation, images of resulting fractions were acquired, and counted for stained (ASC) and unstained (MDA-MB-231) cells. Data are presented as mean ± standard deviation (n = 10). Bar equals 200 µm.

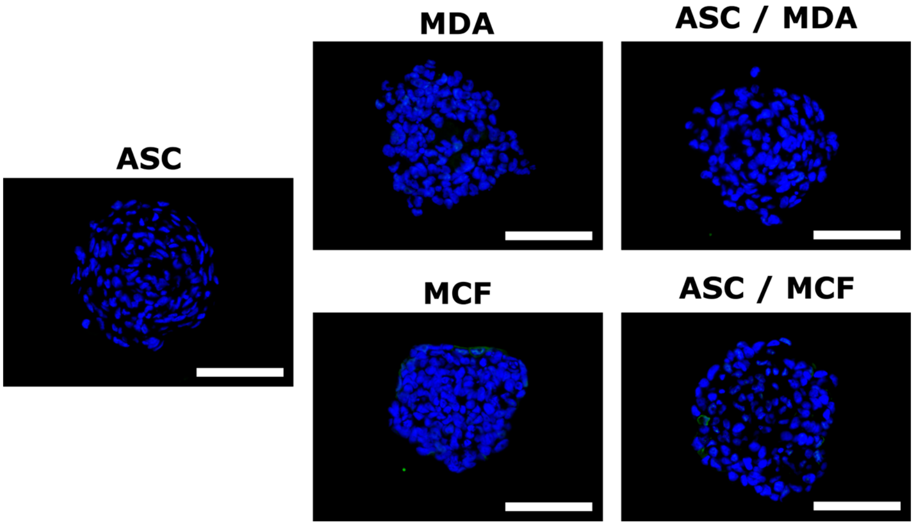

Supplementary Figure S2: Protein expression of CCR5 in mono- and co-culture spheroids of ASC und MDA-MB-231 and MCF-7 after 48 h. Mono- and co-spheroids were immunohistochemically stained for CCR5 (green). Nuclei were stained with DAPI (blue). Bar equals 100 µm.

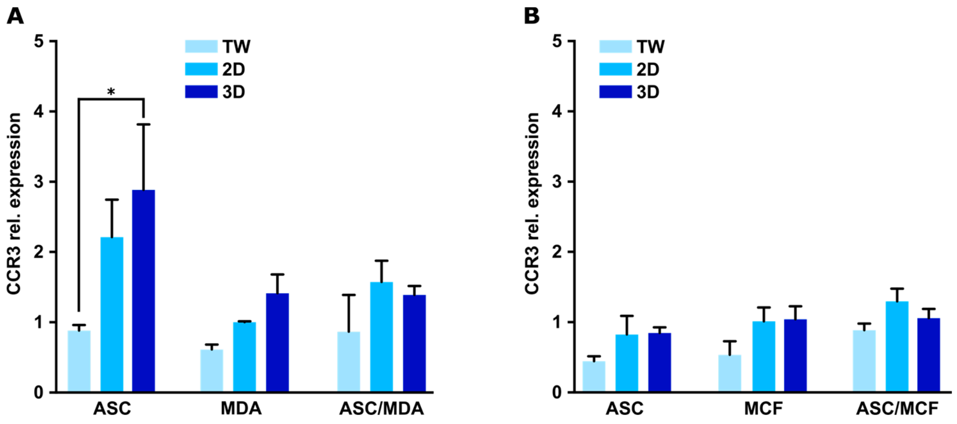

Supplementary Figure S3: CCR3 gene expression in mono- and cocultures of ASCs and MDA-MB-231 or MCF-7 in varying culture systems after 48 h. Gene expression was assessed using qRT-PCR and was normalized to the housekeeping gene EF1α; obtained values were further normalized to standard 2D MDA-MB-231 or MCF-7 monoculture. Data are presented as mean ± standard deviation (n = 3). * indicates statistically significant differences ($p < 0.05$) between culture systems.

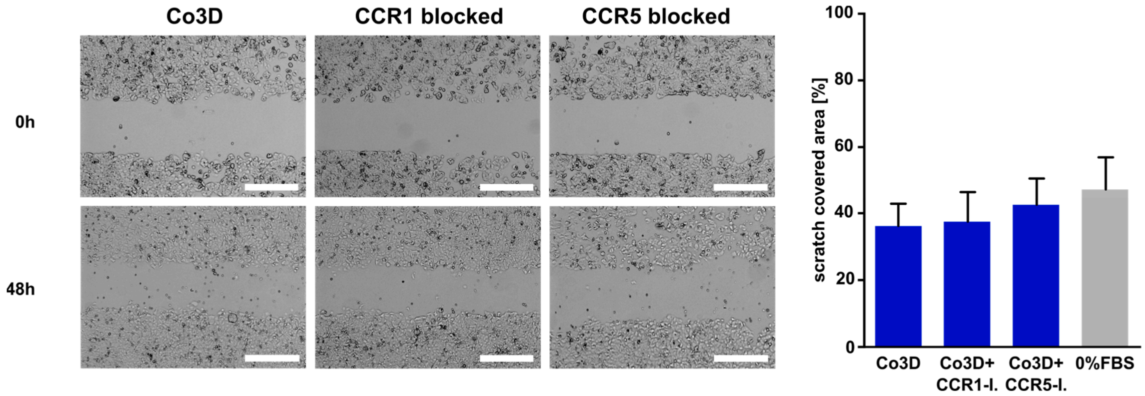

Supplementary Figure S4: Impact of conditioned medium (CM) from ASC/MCF-7 co-spheroids and CCL5 receptor blocking on MCF-7 migration. Migration (scratch) assay of MCF-7 cells in CM of the 3D co-spheroids (Co3D). Specific CCR1 (BX471) and CCR5 (TAK-779) antagonists were added to the conditioned medium (Co3D+CCR5-I, Co3D+CCR1-I) and migration was assessed. Growth medium without FBS served as control (0% FBS). Images were acquired and analyzed as described above. Bar equals 500 µm. Data are presented as mean ± standard deviation (n = 12). * indicates statistically significant differences ($p < 0.05$).

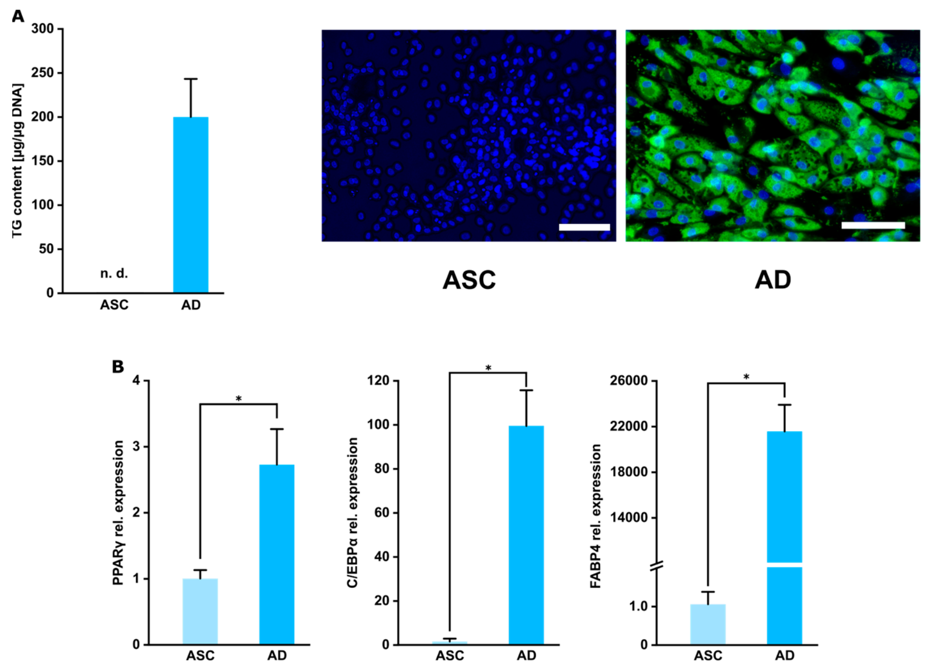

Supplementary Figure S5: Characterization of differentiated adipocytes. (**A**) Intracellular triglyceride content of adipocytes after 14 days of adipogenic differentiation. ASCs were adipogenically differentiated using an adipogenic hormonal cocktail for 14 days. Triglyceride contents in untreated ASCs or adipocytes were normalized to the respective DNA samples (**A**, left). Immunohistochemical staining (perilipin) of accumulated lipid droplets (**A**, right) in untreated ASCs or adipocytes after 14 days. Bar equals 100 µm. (**B**) Adipogenic marker gene expression after 14 days of adipogenic differentiation. Gene expression was assessed using qRT-PCR and was normalized to the housekeeping gene EF1α; obtained values were further normalized to undifferentiated ASCs. Data are presented as mean ± standard deviation (n = 3). * indicates statistically significant differences ($p < 0.05$).

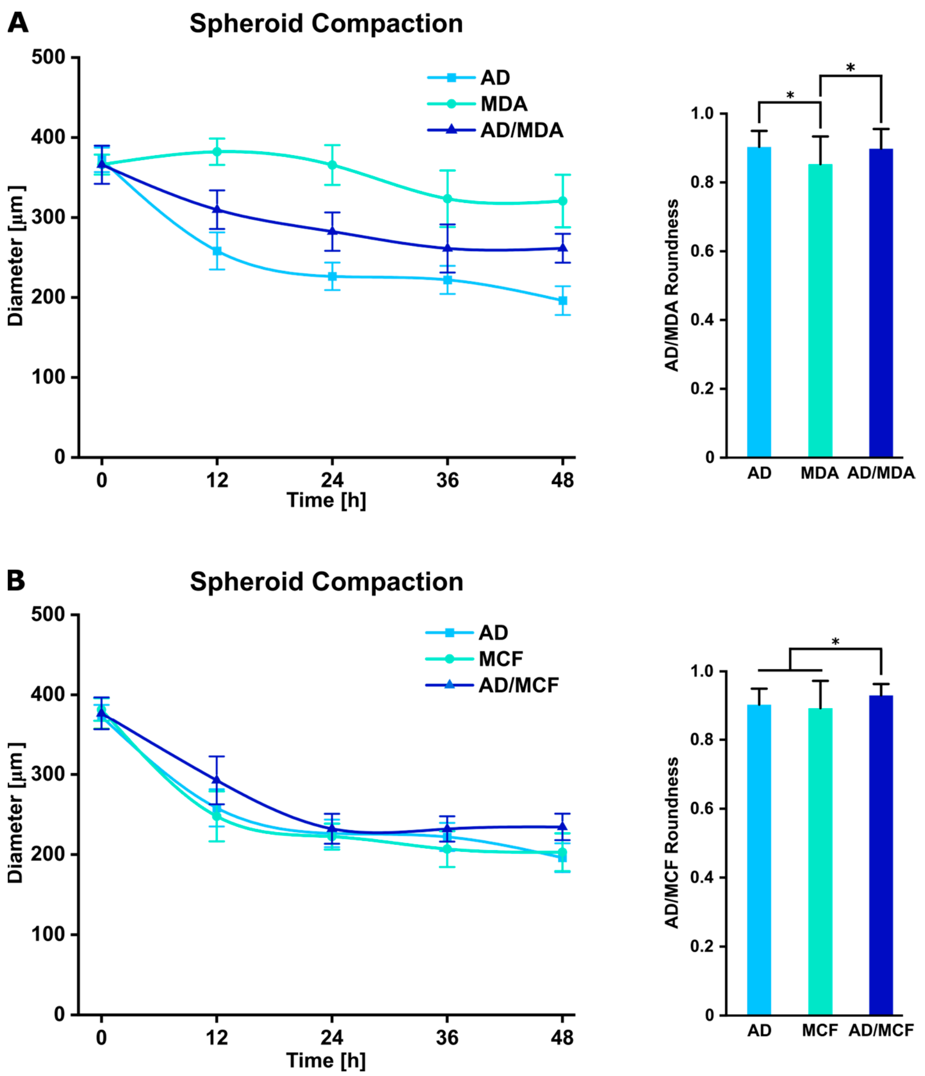

Supplementary Figure S6: Characterization of spheroids containing adipocytes and MDA-MB-231 (A) or MCF-7 (B). Cells were permitted to form spheroids in micromolds for 48 h. For analysis of spheroid compaction, images of spheroids were acquired every 12 h for a total of 48 h and diameter was measured. Roundness values were determined after 48 h using ImageJ. Data are presented as mean ± standard deviation (n = 80). * indicates statistically significant differences ($p < 0.05$).
